# Is percutaneous drainage better than endoscopic drainage in the management of patients with malignant obstructive jaundice? A meta-analysis of RCTs

**DOI:** 10.3389/fonc.2023.1105728

**Published:** 2023-01-30

**Authors:** Cnogwen Bian, Yuan Fang, Jun Xia, Lan Shi, Hanfei Huang, Qiru Xiong, Ruolin Wu, Zhong Zeng

**Affiliations:** ^1^ Department of General Surgery, The First Affiliated Hospital of Kunming Medical University, Kunming, China; ^2^ Department of Surgery, Li Ka Shing Faculty of Medicine, The University of Hong Kong, Hong Kong, Hong Kong SAR, China; ^3^ Department of General Surgery, The First Affiliated Hospital of Anhui Medical University, Hefei, China; ^4^ Department of the Fourth Clinical Medical College, Zhejiang Chinese Medical University, Hangzhou, Zhejiang, China; ^5^ Department of General Surgery, The Second, Affiliated Hospital of Anhui Medical University, Hefei, China; ^6^ Department of Hepatopancreatobiliary Surgery and Organ Transplantation Center, Department of General Surgery, The First Affiliated Hospital of Anhui Medical University, Hefei, China

**Keywords:** ERCP, PTCD, Malignant obstructive jaundice, Procedure-related complication, Meta-analysis

## Abstract

To compare the safety and efficacy of endoscopic retrograde cholangiopancreatography (ERCP) and percutaneous transhepatic cholangial drainage (PTCD) in the treatment of malignant obstructive jaundice, a systematic review and meta-analysis of published studies was undertaken to assess the differences between the two procedures in terms of efficacy and safety. From November 2000 to November 2022, the Embase, PubMed, MEDLINE, and Cochrane databases were searched for randomized controlled trials (RCTs) on the treatment of malignant obstructive jaundice with ERCP or PTCD. Two investigators independently assessed the quality of the included studies and extracted the data. Six RCTs, including 407 patients, were included. The results of the meta-analysis showed that the overall technical success rate in the ERCP group was significantly lower than that in the PTCD group (Z=3.19, P=0.001, OR=0.31 (95% CI: 0.15-0.64)), but with a higher overall procedure-related complication incidence rate (Z=2.57, P=0.01, OR=0.55 (95% CI: 0.34-0.87)). The incidence of procedure-related pancreatitis in the ERCP group was higher than that in the PTCD group (Z=2.80, P=0.005, OR=5.29 (95% CI: 1.65-16.97)), and the differences were statistically significant. No significant difference was observed between the two groups when the clinical efficacy, postoperative cholangitis, and bleeding rate were compared.Both treatments for malignant obstructive jaundice were efficacious and safe. However, the PTCD group had a greater technique success rate and a lower incidence of postoperative pancreatitis.The present meta-analysis has been registered in PROSPERO

## 1 Introduction

Obstructive jaundice is caused by biliary stricture and bile excretion obstruction and is most commonly caused by malignant tumor compression or direct metastasis. Malignant obstructive jaundice (MOJ) can lead to pathophysiological disorders of multiple organ systems throughout the body, including systemic electrolyte imbalance, immune system injury, coagulation disorders, digestive system insufficiency, and malnutrition. If the obstruction cannot be removed in time, it may cause biliary infection, liver and kidney failure, and even death (1, 2). Most patients are diagnosed in the middle or advanced stages of the illness, and the tumors are unresectable. The incidence of radical resection among them is approximately 20% (3, 4), and the remaining patients may only select palliative therapy options, such as biliary drainage (BD).

There are many different types of biliary drainage operations in clinical practice, among which two types of procedures are prevalent: 1. Endoscopic retrograde cholangiopancreatography (ERCP): The endoscope is inserted into the descending part of the duodenum through the duodenal papilla into the bile duct, with the biliary stent placed through the site of the obstruction. ERCP, an effective treatment for obstructive jaundice, drains bile into the body or intestinal tract, quickly drains bile to relieve biliary obstruction and compression, removes jaundice, and improves liver function. 2. Percutaneous transhepatic cholangial drainage (PTCD): This procedure involves inserting an internal or external drainage cannula into the dilated bile duct through the liver under the guidance of X-ray or ultrasound to quickly discharge bile and ameliorate jaundice. With the continuous progression of endoscopic and percutaneous drainage, these procedures have gradually become the most effective methods known to alleviate MOJ; they can effectively reduce bilirubin levels in the blood, improve liver function, improve nutritional status, prolong life expectancy, and thus improve the quality of life, especially for obstructive jaundice with unresectable tumors. Therefore, ERCP or PTCD has become the initial treatment for obstructive jaundice, but the optimal treatment remains controversial.

In this study, we aimed to compare the differences in the technique success rate, clinical efficiency, and incidence of postoperative complications between the two methods through evidence-based medical analysis to evaluate the advantages and disadvantages of the two methods in the treatment of MOJ and to explore the best BD method for patients with MOJ.

## 2 Methods

Based on the guidelines of the Preferred Reporting Items for Systematic Reviews and Meta-Analyses (PRISMA) (5) and Cochrane Collaboration (6), we conducted the study with approval from the Institutional Review Board.

### 2.1 Search strategy and identification of studies

From November 2000 to November 2022, randomized controlled trials on the treatment of malignant obstructive jaundice with ERCP or PTCD were searched in the EMBASE, PubMed, MEDLINE, and Cochrane databases using the same index terms “ERCP, PTCD, PTBD, MOJ; endoscopic retrograde cholangiopancreatography, percutaneous transhepatic cholangial drainage, malignant obstructive jaundice”. The included literature had to be randomized controlled trials. Retrospective controlled trials, unpublished literature, case reports, and reviews were also excluded. Two researchers reviewed all of the literature and abstracts according to the study’s requirements, excluding unqualified literature and reading the full text of any literature that could potentially be included to determine whether it met the inclusion criteria. All disagreements were resolved by discussion.

### 2.2 Inclusion and exclusion criteria

#### 2.2.1 Inclusion criteria

All included investigations were English studies comparing PTCD and ERCP for malignant biliary obstruction. Subjects: Malignant obstructive jaundice is typically clinically diagnosed *via* imaging data as biliary stricture or occlusion caused by a primary or metastatic malignant tumor, such as pancreatic cancer, hilar cholangiocarcinoma, ampullary carcinoma, and other tumors. The patients were informed and agreed to participate in the study and provided written informed consent. Intervention measures in the experimental group: ERCP was used to treat malignant obstructive jaundice. The control group was treated with PTCD.

#### 2.2.2 Exclusion criteria

Studies were excluded if they were nonrandomized controlled studies, incomplete randomized controlled studies, retrospective analysis studies, conference abstracts, complete texts without original data, duplicate reporting studies, letters, or review styles.

### 2.3 Data extraction and assessment of the risk of bias

Data on the publication year, authors, number of subjects, methodological characteristics, and evaluation indices (technique success, clinical efficacy, and procedure-related complications) were extracted. The bias risk assessment tool provided by the Cochrane Library was used to assess the quality of randomized controlled trials by two researchers independently, including the method of random allocation and whether subjects and study implementers and measurement results were blinded. The tool also assesses whether the data are complete and selective reporting of research results and other possible sources of bias. A consensus was reached after discussion when a controversy arose. Otherwise, divergence was resolved by third parties.

### 2.4 Statistical methods

The extracted data were statistically analyzed using the software package Rev Man 5.3. To compare outcomes, the odds ratio (OR) and mean difference (MD) were calculated as effect sizes for dichotomous and continuous variables, respectively, including their combined value and 95% confidence interval (95% CI). A χ2 test was conducted to examine the heterogeneity among the included studies using the inconsistency index (I2) statistic. Heterogeneity was identified as P>0.10, I2>50%, in which a random-effects model was used; otherwise, the fixed-effects model was used for homogeneity, and two-sided P<0.05 was considered statistically significant.

## 3 Results

### 3.1 Study selection and trial characteristics

**Figure 1:**
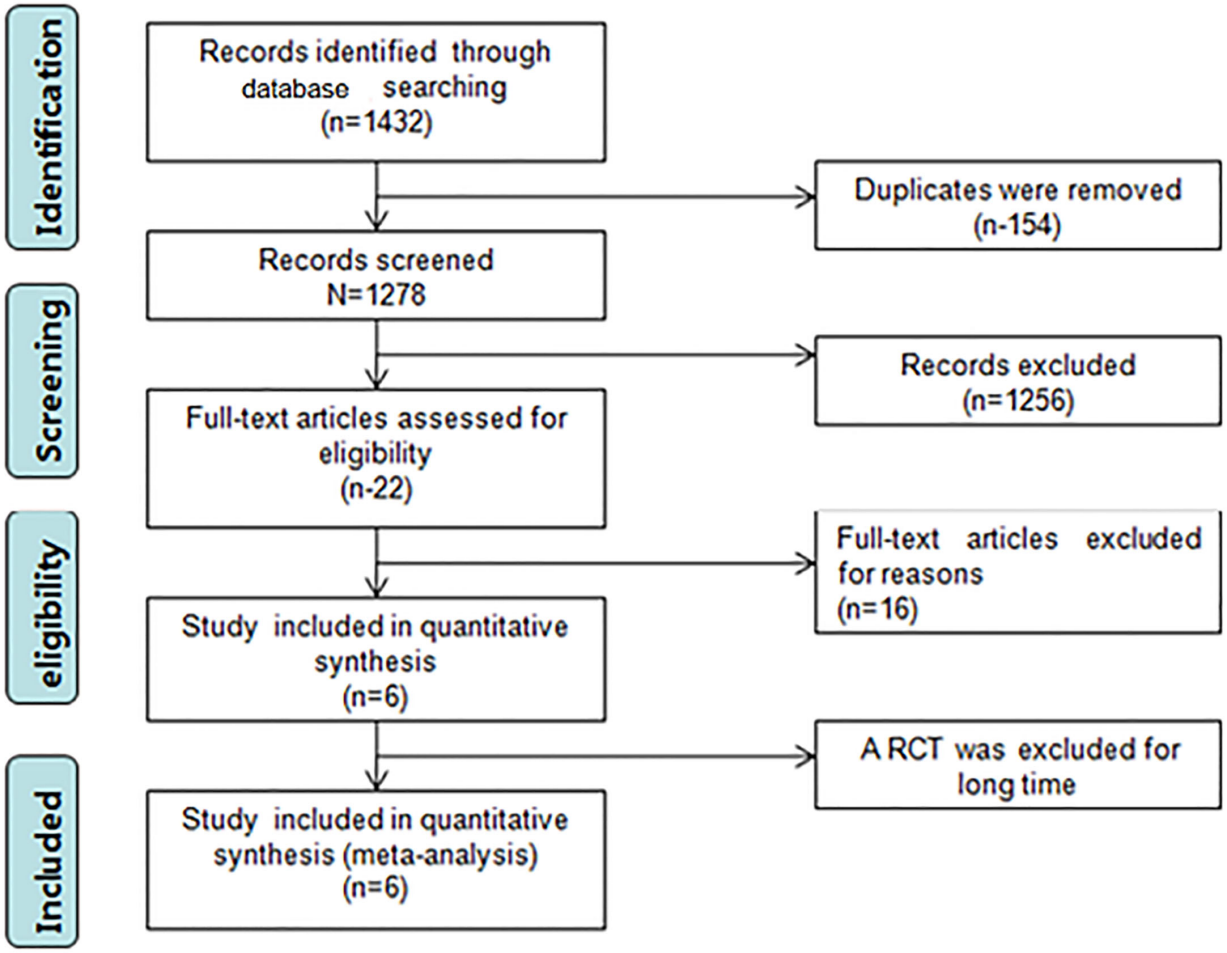
PRISMA flowchart summarizing the study selection process. The enrolled studies represent a total of 6 RCTs and encompass 207 patients with ERCP and 200 patients with PTCD. After quality assessment, all studies were interpreted as high-quality studies. The characteristics of the studies are depicted in **Table 1**.

**Figure 2:**
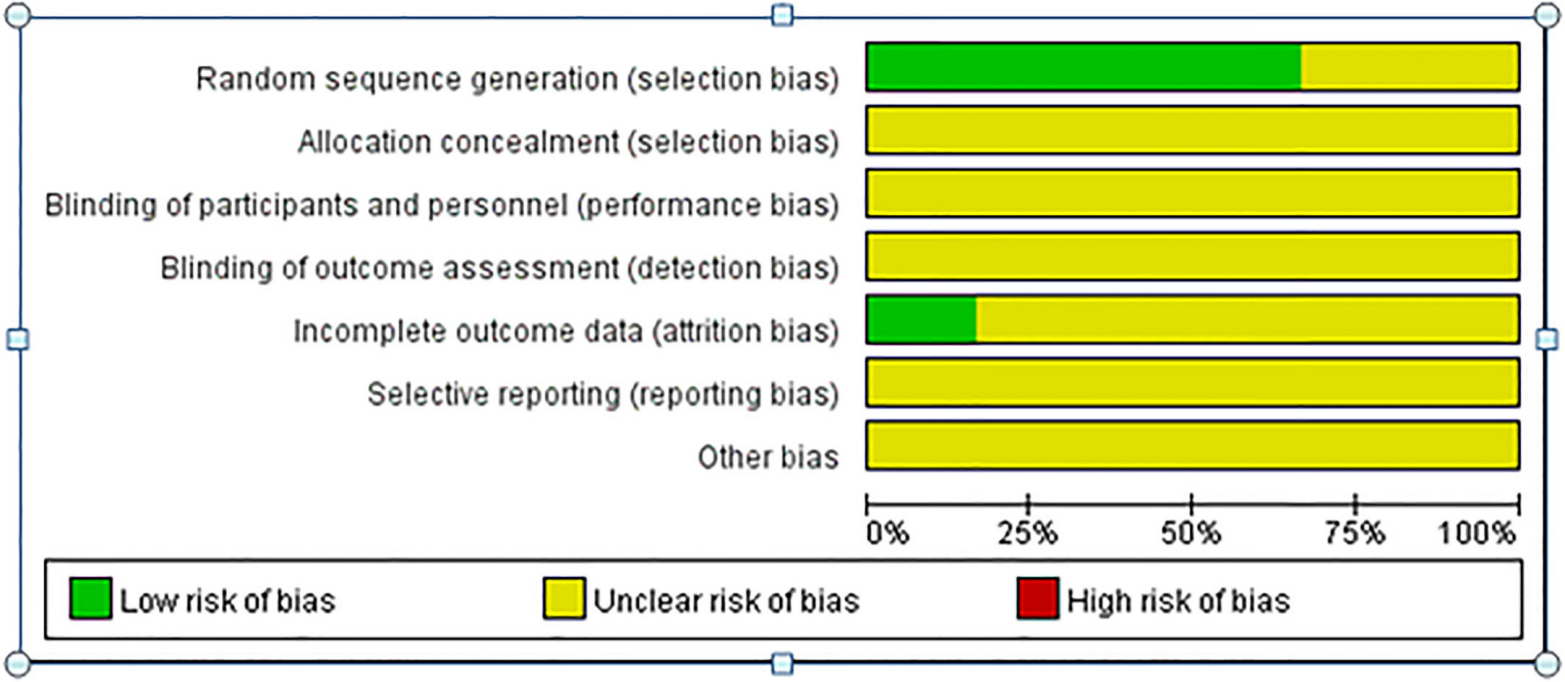
Quality assessment of the enrolled studies.

The search strategy identified 1432 articles, of which 154 duplicate articles were excluded, 1256 irrelevant articles were excluded after reading the titles and abstracts, and 22 articles remained initially. Full texts were assessed for eligibility (conference abstracts and full texts without original data for retrieval, duplicate published studies, letters, non-RCTs, retrospective analyses, and reviews were excluded). Finally, seven articles (7–13) were included in this study. Because of the immature technology recorded in the first RCT paper (12), there would have been significant heterogeneity if it was included, and the analysis would not truly reflect the efficacy and safety of the two procedures; consequently, that RCT was ultimately excluded. **Figure 1** shows the literature search strategy and screening process, and the quality of the included studies is plotted in **Figure 2**. The primary characteristics of the included studies are shown in **Table 1**.

**Table 1:** Main characteristics of the included literature.

Author	Year	Country	Study Design	No. Patients in study		Technique success.		Clinical effectiveness		Complications	
				ERCP	PTCD	ERCP	PTCD	ERCP	PTCD	ERCP	PTCD
GH Bao, et al. (5)	2021	China	RCT	38	31	36	31	34	28	3	7
HM El-Haddad, et al. (6)	2021	Egypt	RCT	34	30	30	30	17	22	4	6
JS Coelen, et al. (7)	2018	Netherlands	RCT	27	27	20	25	17	21	18	17
SS Saluja, et al. (8)	2008	India	RCT	27	27	22	26	11	24	5	14
Virgı ´nia P (11)	2002	Spain	RCT	26	28	15	21	11	20	9	17
XR Sun, et al. (9)	2014	China	RCT	55	57	52	55	49	55	11	3

### 3.2 Technique success

**Figure 3:**
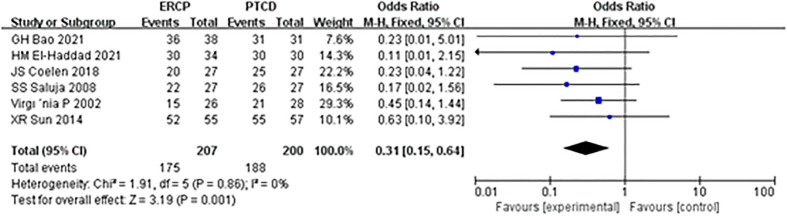
Forest plot comparing the technical success.

The overall technical success rate was reported in all six articles, and there was no heterogeneity among the outcomes; therefore, a statistical analysis was conducted using the fixed effect model. The results of the meta-analysis: Z=3.19, P=0.001, OR=0.31 (95% CI: 0.15-0.64). The difference was statistically significant, and the total success rate of surgery in the PTCD group was significantly higher than that in the ERCP group (**Figure 3**).

### 3.3 Clinical effectiveness

**Figure 4:**
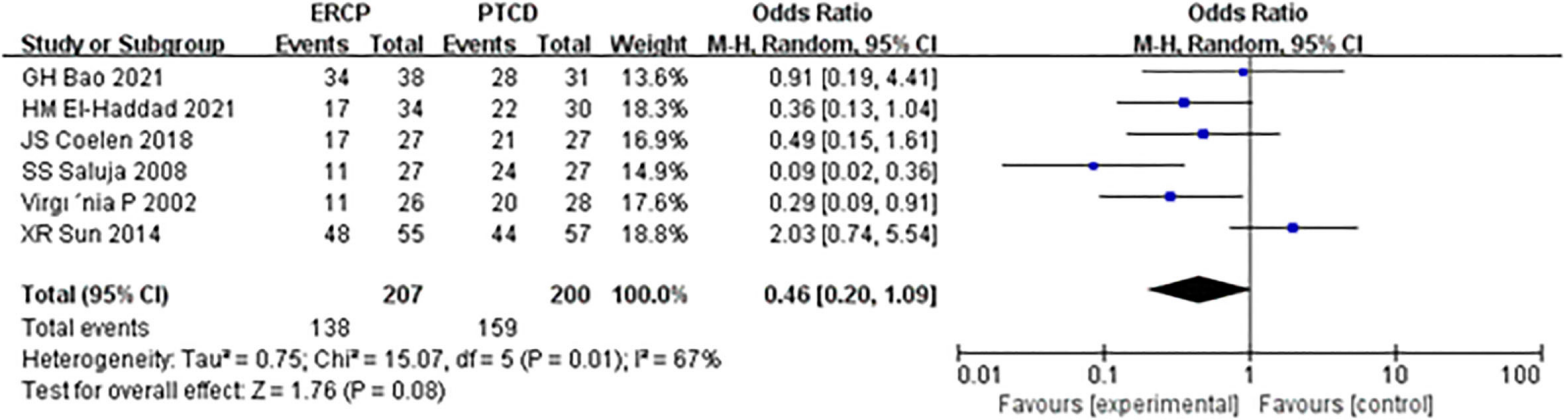
Forest plot comparing the clinical effectiveness.

The total clinical efficacy was reported in six studies, and heterogeneity was observed among the results of each study. The random-effects model was applied, and the results of the meta-analysis were as follows: Z=1.76, P=0.08, OR=0.46 (95% CI: 0.20-1.09), indicating that the difference was not statistically significant, and there was no significant difference in total clinical efficacy between the ERCP and PTCD groups (**Figure 4**).

### 3.4 Procedure-related complications

**Figure 5:**
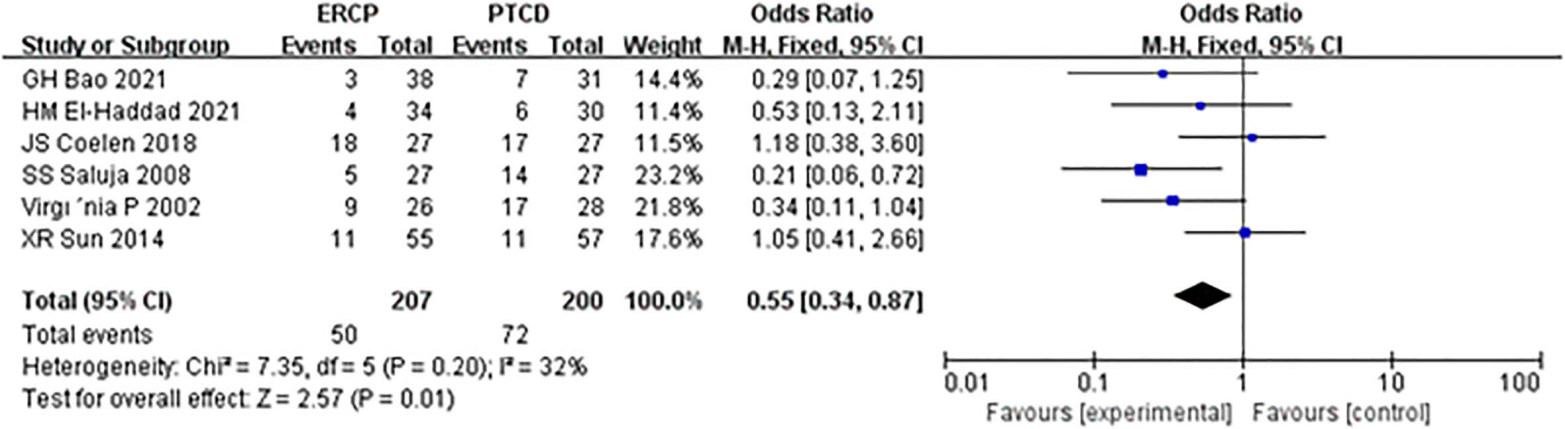
Forest plot comparing the overall complication rate.

The incidence of overall procedure-related complications was described in six studies, and there was no heterogeneity among the results of each study. Statistical analysis was conducted using the fixed-effect model, and the results of the meta-analysis were as follows: Z=2.57, P=0.01, OR=0.55 (95% CI: 0.34-0.87), indicating that there was a significant difference in the total complication rate between the two groups, with the PTCD group having a higher overall complication incidence (**Figure 5**).

### 3.5 Procedure-related cholangitis

**Figure 6:**
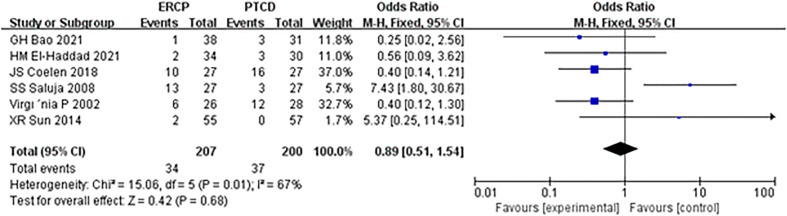
Forest plot comparing the incidence of procedure-related cholangitis.

The incidence of postoperative cholangitis was reported in all six articles, and there was heterogeneity among the results; thus, the random-effects model was used for statistical analysis. The results of the meta-analysis revealed Z=0.21, P=0.83, OR=0.87 (95% CI: 0.24-3.16), and there was no significant difference in the incidence of postoperative cholangitis between the ERCP and PTCD groups (**Figure 6**).

### 3.6 Procedure-related pancreatitis

**Figure 7:**
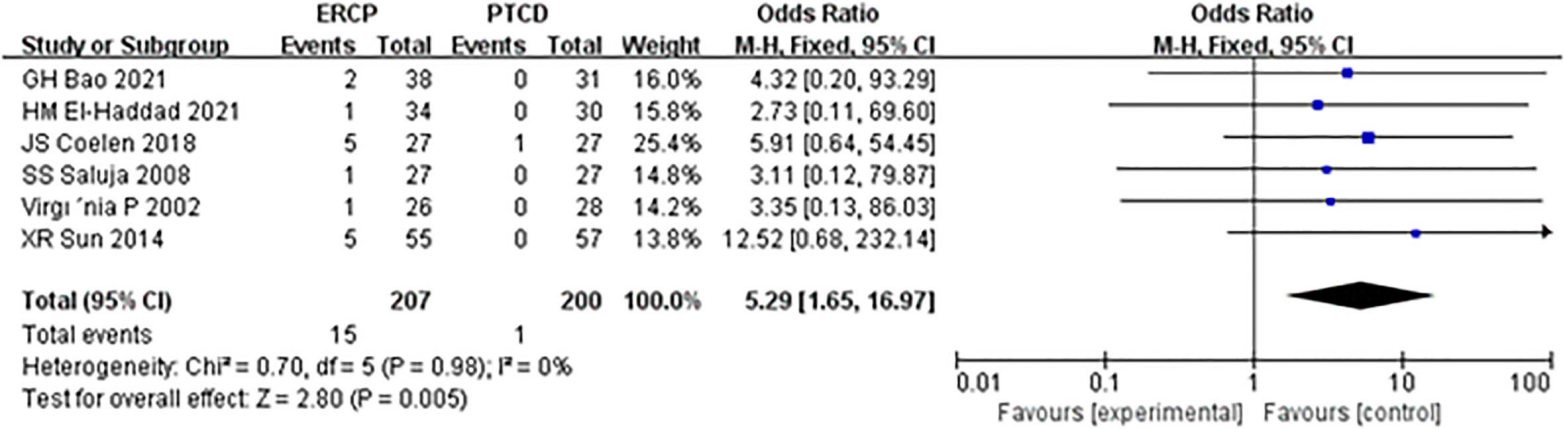
Forest plot comparing the incidence of procedure-related pancreatitis.

Procedure-related pancreatitis was reported in all six articles, and there was no heterogeneity among the results; therefore, statistical analysis was conducted using the fixed-effect model. The results of the meta-analysis were as follows: Z=2.80, P=0.005, OR=5.29 (95% CI: 1.65-16.97). The difference was statistically significant, and the incidence of postoperative pancreatitis in the ERCP group was significantly higher than that in the PTCD group (**Figure 7**).

### 3.7 Procedure-related hemorrhage

**Figure 8:**
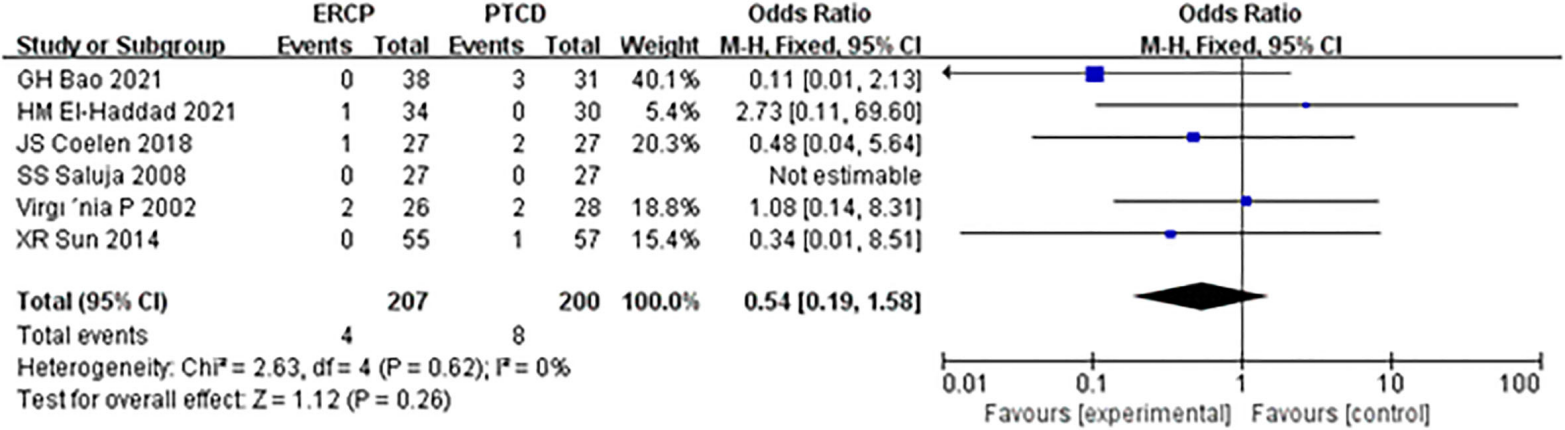
Forest plot comparing the incidence of procedure-related hemorrhage.

There was no heterogeneity among the results; therefore, a fixed-effects model was used for statistical analysis. The results of meta-analysis: Z=1.90, P=0.26, OR=0.54 (95% CI: 0.19-1.58). The difference was statistically significant, and there was no significant difference in the postoperative bleeding rate between the ERCP and PTCD groups (**Figure 8**).

### 3.8 Publication bias

**Figure 9:**
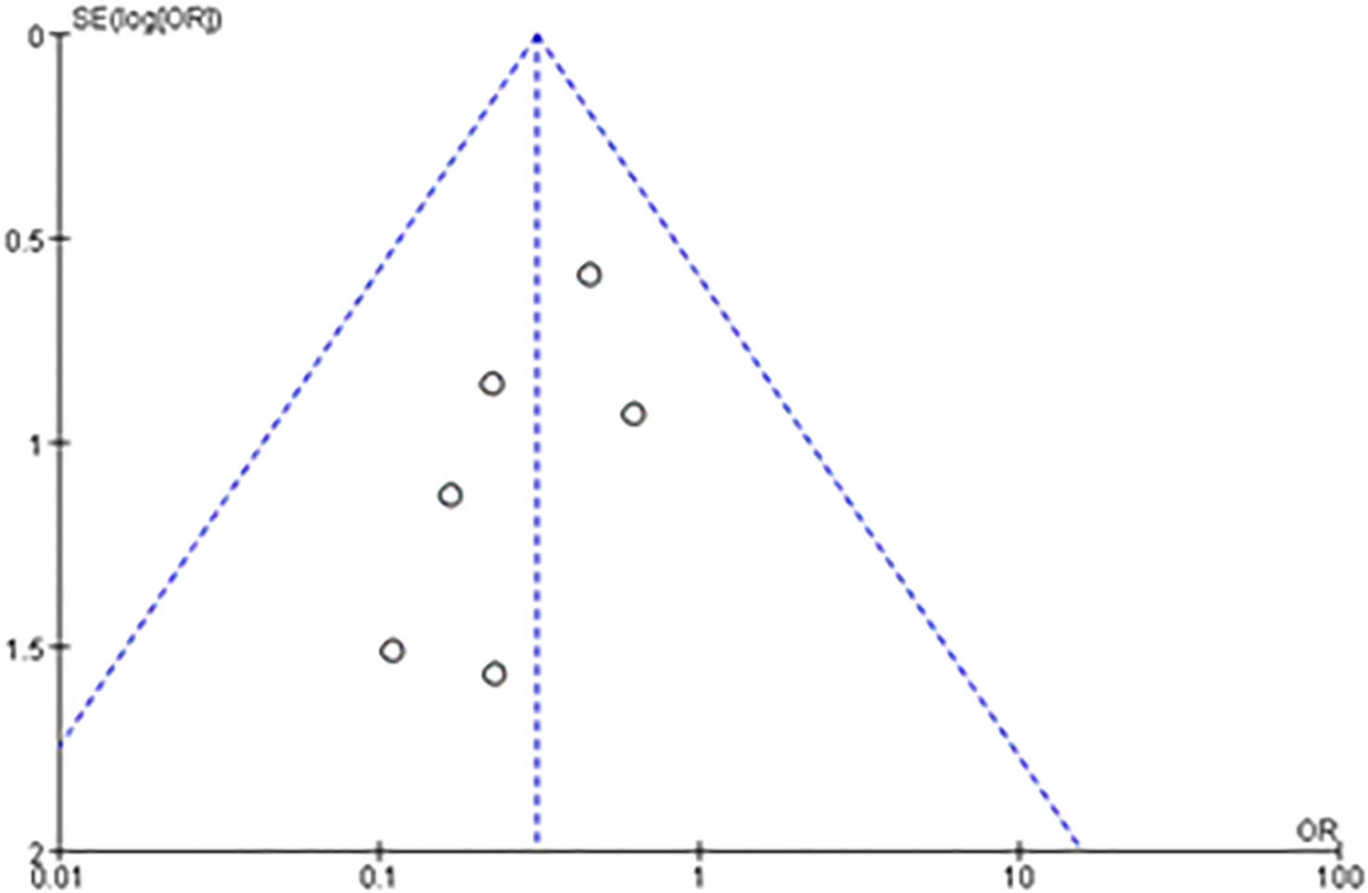
Funnel plot evaluating publication bias for technical success.

Publication bias analysis based on a funnel plot of technique success. No publication bias was detected with the observed indicators (**Figure 9**).

### 3.9 Sensitivity analysis

Sensitivity analysis is a crucial component of meta-analysis because it determines the overall credibility of the observed results. The results can be considered reliable if they remain consistent across sensitivity analyses. A meta-analysis of the remaining studies was conducted to assess the stability of the results. Individual investigations were eliminated item by item using a sensitivity analysis. After excluding each study and reintegrating the effect values, all were within the CI. There was no significant difference before removal (I^2^ = 0), showing that the sensitivity of the included literature was low and that the results of this analysis were stable.

## 4 Discussion

The methods of biliary drainage have been changing with the advancement of endoscopic technology, and PTCD became a prevalent technique in the late 1980s owing to its milder trauma, fewer comprehensive indications, and better economic benefits. PTCD helps restore physiological continuity to the biliary tract *in situ* and provides rapid relief of symptoms. Most patients with obstructive jaundice are treated with PTCD. Although the effect was significant and the prognosis could be improved, the incidence of postprocedural complications was still relatively higher (14, 15). With the improvement and availability of endoscopic technology, PTCD has been gradually replaced by endoscopic drainage (12, 16). However, such technical alternatives lack the support of EBM evidence from RCTs, that is, large-scale data on safety and efficacy from RCTs. Indeed, PTCD may increase the risk of local recurrence and metastasis (17).

In contrast, bile outflow may negatively affect digestive and liver functions. Therefore, some guidelines recommend ERCP as the preferred treatment for malignant obstructive jaundice (18). ERCP is more suitable for patients with physiological characteristics and can better restore the physiological drainage function of bile, improve quality of life, and relieve and delay liver failure.

### 4.1 Technique success and clinical effectiveness

This meta-analysis favors PTCD over ERCP for achieving satisfactory technical success as initial treatment in patients with MOJ. Otherwise, the two treatments had the same effectiveness in biliary drainage. A study (19) reported that the ERCP failure rate is approximately 10%, and the reasons for failure include immature techniques, ambiguous identification of the duodenal papilla, anatomical variation, and severe biliary tract stricture or occlusion caused by malignant obstruction. In comparison, PTCD has a higher procedure success rate than ERCP and can be recommended as the first treatment or remedy after ERCP treatment failure. Clinical effectiveness refers to the improvement in jaundice due to biliary drainage. A comprehensive comparison showed that both treatment methods can effectively decompress malignant biliary obstruction and drain bile. There was no statistically significant difference between the two groups in the clinical efficacy of the procedure for malignant obstructive jaundice (P=0.08). A larger-scale study (20) found that patients with morbidities of high obstruction, biliary sepsis, and liver function with a lower Child‒Pugh classification would have poorer drainage effect, regardless of the difference in the patients’ age, sex, diagnosis, number of stents, obstruction, bile duct diameter, abdominal cavity effusion time, intrahepatic lesion, lymph node metastasis, and distant metastasis. Except for these factors, the reason for the same clinical efficacy in the PTCD group following a higher technique success rate could be explained by the fact that ERCP has a better effect on bile drainage. Internal bile drainage is more favorable for bile acid excretion (21). Oral administration of the lost bile from PTCD significantly shortened the time for total bilirubin to return to normal levels in the blood (22). In addition, the definition of clinical efficacy varied. For example, clinical effectiveness was defined as a 50% reduction in the serum total bilirubin level. In the study by Bao et al. (7), the time of decline was defined as less than two weeks, and in the study by Hany et al. (8), clinical effectiveness was defined as a 50% reduction in the serum total bilirubin level within ten days.

### 4.2 Advent effects

In this meta-analysis, ERCP was associated with fewer overall postprocedural adverse events and more procedure-related pancreatitis than PTCD, which is considered a prognostic factor in patients and a reference strategy in the management of MOJ. Mild complications affect the clinical efficacy in patients, while serious complications may cause disease progression or even lead to the death of patients. The mortality rates associated with ERCP and PTCD have been reported to be 0.1% and 2%, respectively (23, 24). In addition to the reasons for the operation itself, the experience of the operator and whether the operator has received systematic training are also correlated with the occurrence of postoperative complications (25). Short-term complications of ERCP and PTCD mainly include biliary infection, acute pancreatitis, hemorrhage biliary leakage, liver abscess, duodenal perforation, and pneumothorax, with an overall complication rate of 10% (26). In this study, there was a significant difference in the total incidence of postprocedural complications between the ERCP and PTCD groups (P=0.01), which differs from the results of another meta-analysis (27) published in 2017. There was an insignificant difference between the two groups, given that most of the included studies were retrospective. Postprocedural pancreatitis is a common complication of endoscopic retrograde cholangiopancreatography (ERCP). The incidence of ERCP-associated pancreatitis reported in the literature (28) is 2.1%-24.4%, and its high-risk factors include repeated intubation, incision of Oddi’s sphincter, and accidental insertion of the main pancreatic duct (29). Subgroup analysis of the included studies showed that the incidence of postoperative pancreatitis in the ERCP group was significantly higher than that in the PTCD group, and the difference was statistically significant. Both the PTCD and ERCP groups were prone to cholangitis, and biliary obstruction was a high-risk factor for cholangitis. In addition, blockage of the drainage stent, stent displacement, and poor drainage effects are common reasons. However, there was no significant difference in the incidence of postoperative cholangitis between the two groups in this study (P=0.83). It was (30, 31) reported that operative bleeding after ERCP and PTCD was 1.6% and 2-3%, respectively. In this study, there was no significant difference in the bleeding rate between the two groups (P=0.26), which was inconsistent with another meta-analysis (32) and may be related to the small sample size and the need for a large RCT sample.

### 4.4 Strengths and limitations

This is the first meta-analysis to compare the efficacy and safety of ERCP with PTCD management of biliary obstruction based on definite RCTs. We systematically evaluated the short-term efficacy and safety of ERCP and PTCD for malignant obstructive jaundice in 6 RCT studies. However, there are still some shortcomings because the inherent limitations of the meta-analysis and the included studies may have weakened our analysis. We could not evaluate the long-term efficacy and safety because such data on 30-day mortality were only provided in one paper. In addition, due to such limitations, we could not analyze the efficacy and safety of different types of procedures. Additionally, there was heterogeneity in a few observation indicators in this study, attributed to the technical variance of operators in different institutions and long time spans, and our comparative analysis of specific complication rates and mortality was limited by the small sample size. Despite these limitations, we believe that our assessment is reliable for comparing the effectiveness and safety of the two methods.

## 5 Conclusion

Based on the available information and the acknowledged limitations of the datasets included in the present study, which incorporated data from 6 RCT studies that included more than 407 patients, the results of this meta-analysis suggest that PTCD is associated with more procedure-related and postoperative complications than ERCP. With regard to similar clinical efficacy, we recommend ERCP as the initial decompression of malignant biliary obstruction. In addition, both methods are technically demanding operations, and we recommend that unskilled surgeons perform them under supervision to ensure clinical safety.

## Author contributions

CB and JX performed the search and drafted the manuscript. YF and HH performed the data extraction and analyzed the data. ZZ and QX designed the study and amended the original draft. All authors contributed to the article and approved the submitted version.
